# Chronic migraine: treatability, refractoriness, pseudo-refractoriness

**DOI:** 10.1186/1129-2377-16-S1-A39

**Published:** 2015-09-28

**Authors:** Piero Barbanti, Cinzia Aurilia, Gabriella Egeo, Luisa Fofi, Serena Piroso

**Affiliations:** Headache and Pain Unit, Department of Neurological Motor and Sensorial Sciences, IRCCS San Raffaele Pisana, Rome, Italy

Chronic migraine (CM), a highly disabling condition affecting 2-3% of the general population, represents a difficult-to-treat disorder for its unclear pathophysiology, complex comorbidities, and disappointing response to available pharmacological treatments[1]. High quality evidence (≥2 RCTs) recommends the prophylactic use of onabotulinum toxin A (155-195 IU) and topiramate (100 mg) in CM, while lower quality evidence (1 RCT) supports the treatment with sodium valproate (800-1500 mg), gabapentin (2400 mg) and tizanidine (18 mg)[2]. Amitriptyline, memantine, zonisamide and pregabalin may also be of help in CM but their use has been suggested only in open studies[2]. CM patients may show poor or no response to preventative therapies. The consensus statement of the European Headache Federation (EHF) defines CM *refractory* to treatment (rCM) when it does not respond to adequate dosages of at least 3 drugs from the classes of beta-blockers, anticonvulsants, tricyclics, onabotulinum toxin A and others (e.g., flunarizine, candesartan) for at least 3 months each, in absence of medication overuse[3]. This rCM definition has been questioned by some authors who stressed the need of using drugs from different classes, not limited to 3, before making rCM diagnosis[4]. Labeling a patient as affected by rCM may profoundly modify his/her life with heavy psychological, social, work and medico-legal consequences, potentially leading to expensive and still unsatisfying surgical procedures such as occipital nerve stimulation[5]. We point out the risk that the current rCM EHF definition could indeed also include *pseudo-refractory* CM patients, due to potential bias: firstly, a significant proportion of CM patients may spontaneously reverse to episodic migraine, as clearly evidenced in population-based study[6]; secondly, rCM patients may present underlying psychiatric disturbances (e.g., personality disorders) not easily recognized, classified and treated by headache specialists; thirdly, rCM diagnosis could be biased by unproven evidence as current rCM criteria do not specify who should attest patient's previous headache history (theoretically self-reported or stated by unqualified physicians). We suggest that 1) rCM is probably more rare than presently stated; 2) before formulating a diagnosis of rCM, psychiatric disorders should be carefully ruled out by appropriate and thorough psychiatric investigations; 3) when assessing CM patient's past medical history, only clinical data coming from certified headache centers should be considered; 4) CM patients should be followed up for an adequate period of time before making a definite rCM diagnosis.
